# The development, validity, and reliability of the Researcher Investment Tool

**DOI:** 10.1017/cts.2024.673

**Published:** 2025-08-11

**Authors:** Brenda M. Joly, Carolyn Gray, Kassandra Cousineau, Karen Pearson, Valerie S. Harder

**Affiliations:** 1 Public Health Program, Muskie School of Public Service, University of Southern Maine, Portland, USA; 2 Catherine Cutler Institute, Muskie School of Public Service, University of Southern Maine, Portland, USA; 3 Department of Pediatrics and Vermont Child Health Improvement Program, The Robert Larner, M.D. College of Medicine, University of Vermont, Burlington, USA

**Keywords:** Measurement tool, metrics, researcher investment, survey, evaluation

## Abstract

**Background:**

Over the last two decades, there have been significant investments designed to advance clinical and translational research (CTR) with an emphasis on supporting early career investigators and building a cadre of skilled researchers. Despite the investments, there are no comprehensive measurement tools to track individual-level progress along the research continuum as supports are put in place.

**Objective:**

The Researcher Investment Tool (RIT) is a novel tool that was created to provide a consistent approach for measuring individual-level changes in the research career trajectory of investigators receiving support from CTR programs.

**Methods:**

The RIT is a 90-item questionnaire, with eight domains and four sub-domains, designed to measure a researcher’s experiences and perceptions. Several rounds of testing were conducted to assess the tool’s face and content validity as well as the internal consistency and test-retest reliability.

**Results:**

Psychometric testing revealed strong content validity and good internal consistency with Cronbach’s alpha coefficients ranging from 0.85 to 0.97 across all domains. Test-retest reliability results also revealed stability in the domain measures over time with Pearson’s correlation coefficients ranging from 0.70 to 0.98 for all but one domain (.53).

**Conclusions:**

This novel RIT may be useful to evaluators when measuring the impact of investments designed to support early career clinical and translational researchers.

## Introduction

Over the last two decades, the National Institutes of Health (NIH) has made significant contributions to advance clinical and translational research (CTR) through its funding and leadership. Awards such as the Clinical and Translational Science Awards (CTSAs) have helped to foster and support new research by building a cadre of clinical and translational scientists. This network currently includes over 60 leading medical organizations across the United States (US) [1]. Programs like the Institutional Development Award (IDeA) Networks for Clinical and Translational Research (IDeA-CTR), established in 1993, currently support 13 statewide or regional competitive awards among states with historically low NIH funding success [2]. A hallmark feature of these NIH grants is the emphasis on evaluation with a focus on demonstrating the value of investments.

There are several theoretical approaches, tools, and metrics for assessing CTSA and CTR initiatives [3–9]. Current evaluation approaches rely on a range of data collection techniques and sources including, but not limited to, self-reported data, document reviews (e.g., CVs), surveys, administrative data, inventories, bibliometric measures, program tracking tools, and network analysis tools [3]. Yet there are few validated survey instruments that provide individual-level measures, and among the currently published approaches, there is no single tool or data source that captures the full range of individual-level experiences, research supports, and investments to assess changes in one’s research career development, accomplishments, and trajectory [3]. Most qualitative and quantitative data collection tools at the individual-level focus on specific areas such as research skills, activities, or capacity [10–15], research benefits or impacts [16–18], research productivity [19–21], research funding or supports [10,22–27], and research infrastructure, operations or culture [10,15,22]. The varied approaches limit the ability to fully understand potential changes and the factors linked to research success and independence among clinical and translational scientists across the US. There is a need to rigorously measure changes that occur, particularly as early-stage investigators move along the research continuum to achieve research independence and prominence, as well as what changes occur over time as individualized supports (e.g., pilot funding, mentorship, and training) are put in place to foster investigators’ research.

Nationally published guidelines for the evaluation of CTR initiatives funded by the NIH highlight the need for innovative approaches and standardized data collection efforts that generate site-specific findings and allow aggregation across institutions [28]. Moreover, CTSAs have been encouraged to develop evaluations that are prospective and evaluators have been called on to implement “cutting-edge approaches” and to “gain efficiencies” by sharing resources across sites [28]. In response to the guidelines, we propose a new tool, the Researcher Investment Tool (RIT), built off prior evaluation work[3] and designed to measure individual researcher experience and support received throughout one’s research career. Evaluators may use the RIT to measure changes in researchers’ experiences and support over time based on investments provided through CTSA, CTR, and similar programs. The purpose of this article is to describe the development and psychometric characteristics of this novel RIT.

## Methods

### Tool development

The RIT was developed by the NNE-CTR Tracking and Evaluation Core based on a review of the literature including existing tools, measures, and approaches designed to assess the experiences, activities, outputs, and impact of an individual’s research. This narrative review has been reported elsewhere and included a total of 136 publications. The review segmented the literature into the following three areas:

1. **Frameworks, models, or approaches with no underlying measures.** The theoretical constructs and the corresponding descriptions or definitions were recorded.
2. **Measures based on primary data collection.** All individual items from available surveys, evaluation forms, focus groups, and interview protocols were captured. The response options and reviewer notes about the data collection process and participants were also recorded.
3. **Measures/indices based on secondary data (e.g., bibliometric measures)**. All existing bibliometric measures were reviewed to include the measure name, definition, focus, and type (e.g., individual or organizational), as well as the date published. Administrative sources of data were also reviewed for type, measures, and data collection details (e.g., review of curriculum vitae).

All abstracted data were recorded in a shared spreadsheet with separate tabs based on the three categories listed above. Individual measures or items were placed into a focus area drawn from the literature and identified by the lead evaluator (e.g, research publications, skills or competencies ) and linked to their citation. All items were assessed for frequency, similarities, and differences in their use.

Overall, the review revealed widespread variation, a lack of comprehensive tools, and a focus on productivity measures (e.g., publications and funding) and bibliometric measures. Given the nature of CTR awards, the evaluation team used the results of the review to identify prominent domains and underlying measures as well as domains and measures specific to clinical and translational research efforts including mentorship, community engagement, research collaboration, and institutional support. The tool was drafted by the lead evaluator and all citations for each item were recorded. New items were created by the lead evaluator, vetted by the evaluation team, and included in the initial cognitive testing phase conducted with researchers who were senior-level researchers serving in a CTR leadership role.

### Characteristics of the tool

The RIT includes a 90-item questionnaire with two sections that measure a researcher’s experiences and perceptions through a five-point scale. Section one measures experience using a Likert-type response ranging from “1 = no experience” to “5 = extensive experience.” Section two measures researcher perceptions based on a similar Likert-type scale ranging from “1 = not at all” to *“*5 = a great extent.” There is also an optional background section that captures demographic information as well as research role and involvement. On average, it takes 10–15 minutes to complete.

**Figure 1.**
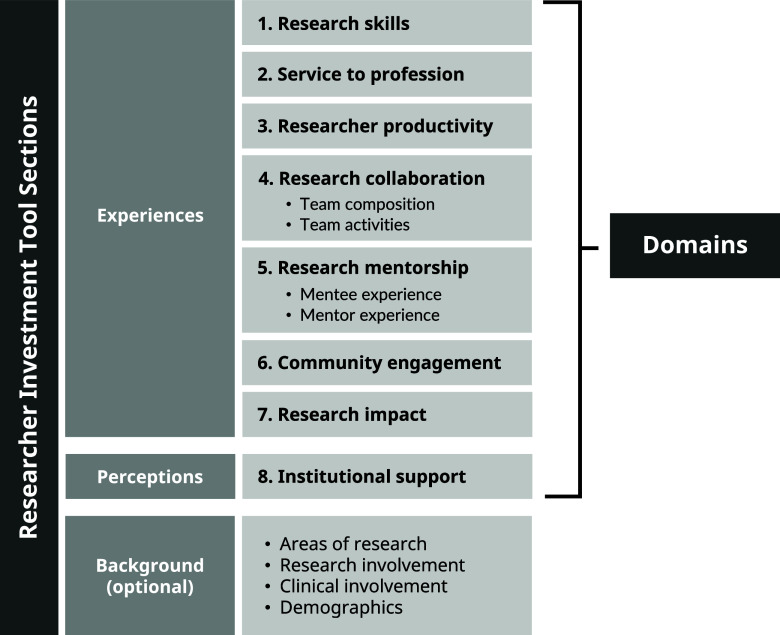
Researcher Investment Tool domains.

As seen in Figure 1, the first two sections of the RIT include eight domains: 1) research skills, 2) service to profession, 3) research productivity, 4) research collaboration, 5) research mentorship, 6) community engagement, 7) research impact, and 8) researcher perceptions of institutional support. Additionally, the research collaboration domain contains two sub-domains, team composition and team collaboration, and the research mentorship domain contains two sub-domains, mentee experience and mentor experience. The items in each domain reflect existing measures compiled from the literature as well as new items added by the NNE-CTR Tracking and Evaluation Core to more fully capture the underlying construct and priorities of clinical and translational research efforts.

### Researcher investment tool domains

#### Research skills

This domain is defined as the experiences investigators have in conducting activities related to research. There are 12 items that reflect the various skills or competencies researchers rely on, including the conceptualization of a research project [14,29], proposal or grant writing [14,22], regulatory compliance and submission of ethics applications [22,30], research management and oversight[29,31] the collection, management, and analyses of data [32], and the dissemination of research findings including reports and presentations [29,32]. Our team added one item to assess overall experience related to participating in someone else’s research, given the focus on new or early-stage investigators.

#### Service to the profession

This 13-item domain reflects the experiences investigators have in participating in research-related service activities. The activities may be self-directed or a result of a personal invitation by others. The service activities focus on teaching [11,22], mentoring [33], serving as a reviewer[11], contributing to new guidelines [11], overseeing research [29], participating in national advisory groups or other committees [34], serving as a peer reviewer for manuscripts and grant proposals [11], serving in an editorial role [35], and training students or postgraduates [36]. Additional items were added based on reviews by senior-level researchers serving in a CTR leadership role and a desire to capture common activities that typically occur by invitation as one’s research becomes more visible and one’s reputation is enhanced due to their work (e.g., presenting research at a national meeting, by invitation).

#### Researcher productivity

This 16-item domain draws on some of the most common measures reported in the literature. Productivity measures are frequently used to track individual-level research outputs and investments. The RIT includes items measuring the experiences investigators have in securing funding, contributing to scientific knowledge, and influencing future research, policies, and practices. The tool measures the type and source of research funding [20,27,37–47], including specific NIH support [48]. The publication metrics, type of publications, and research citations[14,16,22,38] were included to assess the reach of an individual’s research and items reflecting the products[49] or translation of research[16,42] were also included in the RIT. Our team added two items: one to reflect internal funding, an indicator of early-stage support relevant to CTRs, and another to capture inquiries researchers may receive about their work, a common occurrence as investigators’ research becomes more visible.

#### Research collaboration

This domain focuses on the experiences and activities investigators have working with different types of research teams. The 12 items are based on literature exploring two sub-domains: the composition of teams (seven items) and their activities (five items). The literature focuses on research involving multi-disciplines [50–52], a patient voice [53,54], cross-disciplinary approaches [50,52,55–57], and the role of team members [50,56,58]. Our team added three items reflecting multi-institutional efforts and partner engagement to reflect the scope of our CTR initiative.

#### Research mentorship

This 15-item domain is defined as the experience investigators have in receiving tailored support provided by a colleague and their experience providing research mentoring. The items are divided into two sub-domains: mentee experience (seven items) and mentor experience (eight items). Understanding the experiences from both vantage points provides a more comprehensive measure [59]. The items were drawn from expert review as well as prior work focusing on receiving mentorship [25,39,60], engaging and connecting with mentors [33,60,61], learning from mentors[60,62] and mapping out a career path [23]. Key components of mentorship which framed this domain included mentoring students and junior researchers [35,39,63], assisting mentees with developing their own grant proposals and resultant funded projects [33,39,48], helping mentees discover new research opportunities [61,64], and introducing mentees to colleagues in the field. One item was added based on expert review to include the development of a mentor/mentee plan.

#### Community engagement

A core feature of CTR initiatives is the focus on community engagement. This domain includes five items related to the alignment of research with community interests, priorities, or concerns [65–67], efforts to include the community in research [65,66,68], and the dissemination of findings [22,69,70].

#### Research impact

The need to demonstrate the value of research expenditures has been well documented in the literature [5,17,18,71]. Yet, there are few standardized tools in clinical and translational science that measure long-term impact based on an individual’s research. This six-item domain measures the experience investigators have related to the influence of their research efforts. The items focus on research that has influenced health outcomes [6,17,72], policy [72,73], practice or fields of study [6,17,74], and future research[17,74]. Our team added one additional item to reflect contributions to theory.

#### Institutional support

This domain contains 11 items and is separated into a new section due to the emphasis on researcher perceptions versus experience. The domain is defined as the perceptions investigators have regarding the culture, practices, and resources used by an organization to foster research. Items focus on leadership [50,75], seed funding [21,64], designated research time [50], recognition or career advancement [76–78], institutional supports and professional development opportunities [20,60,79,80], multi-disciplinary research [20,56,68,79], and roles, expectations, and rewards[19,46,50,76–78,81].

### Validity testing process

#### Face and content validity

Typically, face and content validity involve reviewing the literature as well as expert review of the items and underlying constructs.[54,82–86] As such, three rounds of validity testing were conducted and revisions to the initial 87-item draft RIT were incorporated after each round of feedback was received. The first round of validity testing involved a comprehensive literature review to abstract and code existing published measures. The second round of validity testing assessed content through cognitive interviewing with two national experts to assess the comprehension, interpretation, and value of each item, the extent to which all items in a domain captured the underlying construct, and the appropriateness of the response options. The third round of testing assessed content validity by surveying 19 senior-level CTR Core Leads and members of the administrative leadership team. The respondents had two weeks to complete the tool, and they were asked to indicate how well each item measured the [named] domain. A five-point star rating was used: one star received a “poor” rating and five stars reflected that an item was an “excellent” match with the [named] domain. Similar to other published efforts, an *a priori* decision was made to remove any items that did not meet our minimum threshold – at least 50% of participants rated the item with four or five stars [6]. In addition to the item rating, respondents were asked to provide open-ended feedback after each domain.

#### Reliability testing process

We tested the reliability (internal consistency and test-retest reliability) of the RIT by administering the tool to 16 staff/faculty (e.g., Research Navigators, CTR grant staff) across two collaborating institutions. The first round took place in February 2024, and fourteen days later, participants were sent the RIT questionnaire for a second round. This time was long enough for them not to have memorized their answers but short enough that none of their answers should have changed. Data were cleaned and analyzed using statistical software package Stata 17.

#### Internal consistency testing

Using the first round of RIT data, we assessed the internal consistency of the eight domains and four sub-domains by calculating Cronbach’s alpha (*α*) coefficients. In addition, we calculated McDonald’s omega (*ω*) coefficients as reported in other similar studies [88,89]. While both approaches can be helpful, Cronbach’s *α* often outperforms McDonald’s *ω* when sample sizes and number of items are small [90]. Domains with a Cronbach’s *α* or McDonald’s *ω* coefficient above 0.70 are generally considered acceptable [91].

#### Test-retest reliability

Using both the first and second rounds of RIT data, we assessed the test-retest reliability of the eight domains and four sub-domains using Pearson’s correlation coefficient (*r*). Similarly, correlations above 0.70 are generally considered strong [92].

## Results

### Cognitive interviewing

Based on feedback during the cognitive interviewing, we removed six items and added 10 items to better assess experience across the following domains: 1) research skills, 2) service to the profession, 3) research productivity, 4) research impact, 5) research collaboration, 6) research mentorship, and 8) research impact. This left us with a 91-item RIT for content validity testing, and we also updated the wording to clarify the domain definitions.

### Content validity

The response rate for the content validity testing was 53% (n = 10/19). Content validity results revealed an overall average across all items of 4.4 on the five-point scale, with “5” being the strongest support. The average rating across domains ranged from 4.0 to 4.8. One item from the research productivity domain was removed based on the *a priori* cutoff. This item focused on developing a research study website. In addition to the removal of one item, resulting in the final 90-item RIT, we incorporated the following changes to reflect the open-ended feedback received during this process. First, we shifted the placement of the community engagement and the research impact domains. Second, we made slight modifications to questions across all eight domains to decrease ambiguity, provide additional examples, specify intent, promote consistency in wording, and ensure items reflected measures related to biomedical and translational research.

### Internal consistency

The response rate for the first round of reliability testing was 100% (*n* = 16). Items five and six within the research productivity domain were dropped from the analysis due to no variability in responses (all responses indicated 1 “No experience”), preventing our ability to run the Cronbach’s *α*. With these items dropped from the analysis, the internal consistency of all eight domains was high (see Table 1), with Cronbach’s *α* ranging from 0.85 (research productivity) to 0.97 (community engagement). As anticipated, the majority of inferences from running McDonald’s *ω* were similar to those of Cronbach’s *α* without notable caveats, ranging from 0.87 (research impact) to 0.97 (community engagement).

**Table 1.** Item counts and reliability results

	Internal Consistency Reliability (*N* = 16)	Test-Retest Reliability (*N* = 12)^2^	
Sections, Domains, and Sub-Domains	Item Count	Cronbach’s Alpha (α) Coefficient	Pearson’s Correlation (*r*) Coefficient
**Researcher Experiences**	77	0.960^1^	0.949**
Research Skills	12	0.892	0.967**
Service to Profession	13	0.903	0.983**
Research Productivity	14	0.849^1^	0.915**
Research Collaboration	12	0.889	0.703*
Team Composition	5	0.710	0.472
Team Collaboration	7	0.900	0.821**
Research Mentorship	15	0.934	0.822**
Mentee Experience	7	0.918	0.623
Mentor Experience	8	0.931	0.865**
Community Engagement	5	0.965	0.847**
Research Impact	6	0.880	0.530
**Researcher Perceptions of Institutional Support**	11	0.964	0.910**

^1^ Items 5 and 6 from research productivity dropped from analysis due to no variability in responses * *p* < 0.05.

^2^ One participant deemed an outlier and dropped from test-retest due to several domains having flipped responses without variation at retest; Three participants did not complete retest. ** *p* < 0.01.

The response rate for the second round of testing was 81% (*n* = 13/16); however, one participant was dropped from analyses due to information bias (they reported to us that their second-round answers were not true after we inquired why there was no variability in numeric values across items within each domain), therefore leaving 75% (*n* = 12/16) of respondents in the final analysis. Test-retest results were strong for seven of the eight domains with significant correlation coefficients greater than 0.70 (*p* < 0.05), while one domain (research impact) had an adequate but non-significant correlation between 0.50 – 0.60.

## Discussion

The RIT provides a first-of-its-kind, standardized, individual-level measure reflecting a broad spectrum of experiences and institutional supports researchers may encounter over time. The RIT domains were drawn from the literature and validated by experts and those involved in clinical and translational research efforts. The psychometric testing revealed strong content validity, acceptable internal consistency across all eight domains, and strong test-retest reliability for nearly all domains, except for the research impact domain. Possible reasons for the lower test-retest reliability include challenges providing one aggregate score when research impact may vary dramatically by project or uncertainty regarding the actual impact of one’s research. Additional refinements to this domain may be needed over time.

As evaluators continue to be called on to document investments in support provided by CTSA, CTR, and other research scholar programs, the need to use valid and reliable measures remains important. This tool may be used over time, for benchmarking, and across institutions and programs to document researcher growth using a consistent approach. It has potential for application in evaluating associations between the scope and magnitude of customized research support. It also can be administered to a defined cohort of scholars to identify strengths and weaknesses based on the experiences reported and the perceived level of support available. The findings can inform future professional development opportunities, mentorship programs, and pilot funding. Given the comprehensive nature of the tool, the RIT also has potential to measure the research productivity and experiences of investigators at all levels, including mid-career investigators. The complete tool has been made available with the intent that it can be widely adopted by others who are seeking to measure the career trajectory of researchers who are receiving tailored, wrap-around support to advance their research path.

### Limitations and next steps

Although the RIT has strong psychometric properties, the tool was developed and tested as part of an evaluation of a CTR initiative. Therefore, the scholars and focus on this program may not be representative across other areas of research. Given the nature of CTR funding, the tool includes a clear focus on community engagement and research collaboration; these domains may not be as relevant to some types of research and interpretations should be made in context.

In addition to the focus areas, the participants may not have been representative and the sample size for the validity and reliability testing was small, with limitations that are consistent with other validation efforts [6]. Future studies testing the tool would benefit from a more robust and diverse sample of researchers and additional advanced statistical techniques to analyze the complex relationship between items and domains. Despite efforts to create domains that reflect the underlying constructs and are exclusive, more research is needed. Future studies that calculate content validity index scores and perform factor analysis, could provide further insight about the items and independence of the domains.

The tool provides a snapshot of cumulative experiences and current supports through a self-report tool that is lengthy due to its comprehensive nature. Repeated measures of the tool will be needed to assess changes over time, and this approach may prove challenging for busy researchers. Finally, additional testing is needed to determine the length of time reasonable changes in each item are likely to be seen and when these changes are best assessed (e.g., years one, three, and five). We plan to conduct longitudinal analyses utilizing the RIT’s ability to measure changes in researcher’s experiences over time and report the RIT’s sensitivity to detect change over multiple time periods. In addition, more work is needed to understand if specific domains could be administered at different time points. For example, are there some domains that warrant inclusion in every administration while others are included in year one and year five? This would shorten the tool and allow scholars more time to gain measurable experience.

## Conclusions

The RIT provides a standardized approach for capturing individual-level measures of a broad spectrum of research experience and supports across eight underlying domains. Given the RIT’s strong psychometric properties, clinical and translational science-based initiatives may consider adopting this tool as part of their broader evaluation efforts. The results can be used to monitor research investments designed to strengthen new- and early-stage clinical and translational research scholars.

## Supporting information

10.1017/cts.2024.673.sm001 Joly et al. supplementary material Joly et al. supplementary material

## Supplementary material

The supplementary material for this article can be found at http://dx.doi.org/10.1017/cts.2024.673.

## References

[1] National Institutes of Health. Clinical and translational science awards (CTSA) program. NIH. 2024. Accessed March 5, 2024. (https://ncats.nih.gov/research/research-activities/ctsa)

[2] National Institute of General Medical Sciences. Institutional development award (IDeA) networks for clinical and translational research (IDeA-CTR). IDeA interactive portfolio dashboard. NIH. 2023. April 11. Accessed March 5, 2024. (https://www.nigms.nih.gov/Research/DRCB/Pages/DRCB-IDeA-Interactive-Portfolio-Dashboard.aspx?awardsby=subprogram&subprgm=IDeA%20-%20CTR)

[3] Center for Leading Innovation and Collaboration. Measures of impact working group: Final report. 2020. (https://clic-ctsa.org/node/6481) February 24. Accessed March 23, 2021.

[4] Centers for Disease Control and Prevention. Framework for program evaluation in public health. MMWR. 1999;48(RR-11):1–58. (http://www.cdc.gov/mmwr/PDF/rr/rr4811.pdf) 10499397

[5] Cruz Rivera S , Kyte DG , Aiyegbusi OL , Keeley TJ , Calvert MJ. Assessing the impact of healthcare research: a systematic review of methodological frameworks. PLoS Med. 2017;14(8):e1002370. doi: 10.1371/journal.pmed.1002370. 28792957 PMC5549933

[6] Dembe AE , Lynch MS , Gugiu PC , Jackson RD. The translational research impact scale: development, construct validity, and reliability testing. Eval Health Prof. 2014;37(1):50–70. doi: 10.1177/0163278713506112. 24085789 PMC4230009

[7] Patel PA , Patel KK , Gopali R , Reddy A , Bogorad D , Bollinger K. The relative citation ratio: examining a novel measure of research productivity among southern academic ophthalmologists. Semin Ophthalmol. 2022;37(2):195–202. doi: 10.1080/08820538.2021.1915341. 34283675

[8] Rollins L , Llewellyn N , Ngaiza M , Nehl E , Carter DR , Sands JM. Using the payback framework to evaluate the outcomes of pilot projects supported by the Georgia clinical and translational science alliance. J Clin Transl Sci. 2021;5(1):1–9. doi: 10.1017/cts.2020.542. PMC8057435 33948270

[9] Vanderbilt University. Flight tracker. 2024. (https://edgeforscholars.vumc.org/information/flight-tracker/) Accessed March 5, 2024.

[10] Brennan SE , McKenzie JE , Turner T , et al. Development and validation of SEER (Seeking, engaging with and evaluating research): a measure of policymakers’ capacity to engage with and use research. Health Res Policy Syst. 2017;15(1):1. doi: 10.1186/s12961-016-0162-8. 28095915 PMC5240393

[11] Caminiti C , Iezzi E , Ghetti C , De’ Angelis G , Ferrari C. A method for measuring individual research productivity in hospitals: development and feasibility. BMC Health Serv Res. 2015;15(1):468. doi: 10.1186/s12913-015-1130-7. 26467208 PMC4607003

[12] Embi PJ , Tsevat J. Commentary: the relative research unit: providing incentives for clinician participation in research activities. Acad Med. 2012;87(1):11–14. doi: 10.1097/ACM.0b013e31823a8d99. 22201633 PMC3914136

[13] Holden L , Pager S , Golenko X , Ware RS. Validation of the research capacity and culture (RCC) tool: measuring RCC at individual, team and organisation levels. Aust J Prim Health. 2012;18(1):62–67. doi: 10.1071/PY10081. 22394664

[14] Smith H , Wright D , Morgan S , Dunleavey J , Moore M. The research spider: a simple method of assessing research experience. Prim Health Care Res Dev. 2002;3(3):139–140. doi: 10.1191/1463423602pc102xx.

[15] The Global Health Network. Using the TDR global competency framework for clinical research: A set of tools to help develop clinical researchers. World Health Organization;2016. Accessed January 26, 2024. (https://media.tghn.org/articles/TDR_Framework_Full_Competency_Tools_20161101_compressed.pdf)

[16] Canadian Academy of Health Sciences, Panel on Return on Investment in Health Research. Making an impact: A preferred framework and indicators to measure returns on investment in health research. Canadian Academy of Health Sciences;2009. Accessed February 9, 2024. (https://www.cahs-acss.ca/wp-content/uploads/2011/09/ROI_FullReport.pdf)

[17] Croxson B , Hanney S , Buxton M. Routine monitoring of performance: what makes health research and development different? J Health Serv Res Policy. 2001;6(4):226–232. doi: 10.1258/1355819011927530. 11685787

[18] Grazier KL , Trochim WM , Dilts DM , Kirk R. Estimating return on investment in translational research: methods and protocols. Eval Health Prof. 2013;36(4):478–491. doi: 10.1177/0163278713499587. 23925706 PMC4084908

[19] Bland CJ , Center BA , Finstad DA , Risbey KR , Staples JG. A theoretical, practical, predictive model of faculty and department research productivity. Acad Med. 2005;80(3):225–237. doi: 10.1097/00001888-200503000-00006. 15734804

[20] Brocato JJ , Mavis B. The research productivity of faculty in family medicine departments at U.S. Medical schools: a national study. Acad Med. 2005;80(3):244–252. doi: 10.1097/00001888-200503000-00008. 15734806

[21] Skinnider MA , Squair JW , Twa DDW , et al. Characteristics and outcomes of Canadian MD/PhD program graduates: a cross-sectional survey. CMAJ Open. 2017;5(2):E308–e314. doi: 10.9778/cmajo.20160152. PMC5498176 28442493

[22] Alison JA , Zafiropoulos B , Heard R. Key factors influencing allied health research capacity in a large Australian metropolitan health district. J Multidiscip Healthc. 2017;10:277–291. doi: 10.2147/JMDH.S142009. 28860795 PMC5558427

[23] Cordrey T , King E , Pilkington E , Gore K , Gustafson O. Exploring research capacity and culture of allied health professionals: a mixed methods evaluation. BMC Health Serv Res. 2022;22(1):85. doi: 10.1186/s12913-022-07480-x. 35039018 PMC8764821

[24] Farrokhyar F , Bianco D , Dao D , et al. Impact of research investment on scientific productivity of junior researchers. Transl Behav Med. 2016;6(4):659–668. doi: 10.1007/s13142-015-0361-9. 27351991 PMC5110485

[25] Goldstein AM , Blair AB , Keswani SG , Gosain A , Morowitz M , Kuo JS , et al. A roadmap for aspiring surgeon-scientists in today’s healthcare environment. Ann Surg. 2019;269(1):66–72. doi: 10.1097/sla.0000000000002840. 29958227 PMC6298819

[26] National Institute of Environmental Health Sciences. Introduction to CareerTrac: Tracking trainees to success. NIH 2019. (https://careertrac.niehs.nih.gov/public/CareerTrac%20Overview%20(May%202019).pdf) May. Accessed February 13, 2023.

[27] Robinson GF , Schwartz LS , DiMeglio LA , Ahluwalia JS , Gabrilove JL. Understanding career success and its contributing factors for clinical and translational investigators. Acad Med. 2016;91(4):570–582. doi: 10.1097/acm.0000000000000979. 26509600 PMC4811729

[28] Trochim WM , Rubio DM , Thomas VG. Evaluation guidelines for the clinical and translational science awards (CTSAs). Clin Transl Sci. 2013;6(4):303–309. doi: 10.1111/cts.12036. 23919366 PMC3931516

[29] Julé A , Furtado T , Boggs L , et al. Developing a globally applicable evidence-informed competency framework to support capacity strengthening in clinical research. BMJ Glob Health. 2017;2(2):e000229. doi: 10.1136/bmjgh-2016-000229. PMC5435268 28589027

[30] Zink HR , Curran JD. Measuring the startup journey and academic productivity of new research faculty through systems engagement, project efficiency, and scientific publication. J Res Adm. Spring. 2020;51(1):32–45.

[31] Saleh M , Naik G , Jester P , et al. Clinical investigator training program (CITP) - a practical and pragmatic approach to conveying clinical investigator competencies and training to busy clinicians. Contemp Clin Trials Commun. 2020;19:100589. doi: 10.1016/j.conctc.2020.100589. 32617432 PMC7322677

[32] Mills BA , Caetano R , Rhea AE. Factor structure of the clinical research appraisal inventory (CRAI). Eval Health Prof. 2014;37(1):71–82. doi: 10.1177/0163278713500303. 23960271 PMC4383464

[33] Bice MR , Hollman A , Ball J , Hollman T. Mentorship: an assessment of faculty scholarly production, mode of doctoral work, and mentorship. Am J Distance Educ. 2022;36(3):208–226. doi: 10.1080/08923647.2021.1941724.

[34] Mason JL , Lei M , Faupel-Badger JM , et al. Outcome evaluation of the national cancer institute career development awards program. J Cancer Educ. 2013;28(1):9–17. doi: 10.1007/s13187-012-0444-y. 23292841 PMC3608862

[35] Finney JW , Amundson EO , Bi X , et al. Evaluating the productivity of VA, NIH, and AHRQ health services research career development awardees. Acad Med. 2016;91(4):563–569. doi: 10.1097/acm.0000000000000982. 26556291

[36] Yan P , Lao Y , Lu Z , et al. Health research capacity of professional and technical personnel in a first-class tertiary hospital in northwest China: multilevel repeated measurement, 2013–2017, a pilot study. Health Res Policy Syst. 2020;18(1):103. doi: 10.1186/s12961-020-00616-7. 32943062 PMC7499869

[37] Akabas M , Brass L , Tartakovsky I. National MD-PhD program outcomes study. Association of American Medical Colleges; 2018. (https://www.aamc.org/data-reports/workforce/report/national-md-phd-program-outcomes-study) Accessed April 6, 2023.

[38] Bawden J , Manouchehri N , Villa-Roel C , Grafstein E , Rowe BH. Important returns on investment: an evaluation of a national research grants competition in emergency medicine. CJEM. 2010;12(1):33–38. doi: 10.1017/s1481803500011994. 20078916

[39] Blanchard RD , Kleppel R , Bianchi DW. The impact of an institutional grant program on the economic, social, and cultural capital of women researchers. J Womens Health. 2019;28(12):1698–1704. doi: 10.1089/jwh.2018.7642. PMC6998046 31259641

[40] Brass LF , Akabas MH. The national MD-phD program outcomes study: relationships between medical specialty, training duration, research effort, and career paths. JCI Insight. 2019;4(19):1–10. doi: 10.1172/jci.insight.133009. PMC6795497 31578310

[41] Dev AT , Kauf TL , Zekry A , et al. Factors influencing the participation of gastroenterologists and hepatologists in clinical research. BMC Health Serv Res. 2008;8:208. doi: 10.1186/1472-6963-8-208. 18842135 PMC2572062

[42] Hsiao CJ , Fresquez AM , Christophers B. Success and the next generation of physician-scientists. J Clin Transl Sci. 2020;4(6):477–479. doi: 10.1017/cts.2020.491. 33948222 PMC8057406

[43] Joss-Moore LA , Lane RH , Rozance PJ , Bird I , Albertine KH. Perinatal research society’s young investigator workshop prepares the next generation of investigators. Reprod Sci. 2022;29(4):1271–1277. doi: 10.1007/s43032-021-00836-4. 35020187 PMC8917055

[44] Mahoney MC , Verma P , Morantz S. Research productivity among recipients of AAFP foundation grants. Ann Fam Med. 2007;5(2):143–145. doi: 10.1370/afm.628. 17389538 PMC1838680

[45] Mavis B , Katz M. Evaluation of a program supporting scholarly productivity for new investigators. Acad Med. 2003;78(7):757–765. doi: 10.1097/00001888-200307000-00020. 12857699

[46] Panettieri RA , Kolls JK , Lazarus S , et al. Impact of a respiratory disease young investigators’ forum on the career development of physician-scientists. ATS Sch. 2020;1(3):243–259. doi: 10.34197/ats-scholar.2019-0018OC. 33870292 PMC8043310

[47] Tesauro GM , Seger YR , Dijoseph L , Schnell JD , Klein WM. Assessing the value of a small grants program for behavioral research in cancer control. Transl Behav Med. 2014;4(1):79–85. doi: 10.1007/s13142-013-0236-x. 24653778 PMC3958595

[48] Sweeney C , Schwartz LS , Toto R , Merchant C , Fair AS , Gabrilove JL. Transition to independence: characteristics and outcomes of mentored career development (KL2) scholars at clinical and translational science award institutions. Acad Med. 2017;92(4):556–562. doi: 10.1097/acm.0000000000001473. 28351069 PMC5373479

[49] Sarli CC , Dubinsky EK , Holmes KL. Beyond citation analysis: a model for assessment of research impact. J Med Libr Assoc. 2010;98(1):17–23. doi: 10.3163/1536-5050.98.1.008. 20098647 PMC2801963

[50] Connors MC , Pacchiano DM , Stein AG , Swartz MI. Building capacity for research and practice: a partnership approach. Future Child. 2021;31(1):119–135.

[51] Libby AM , Hosokawa PW , Fairclough DL , Prochazka AV , Jones PJ , Ginde AA. Grant success for early-career faculty in patient-oriented research: difference-in-differences evaluation of an interdisciplinary mentored research training program. Acad Med. 2016;91(12):1666–1675. doi: 10.1097/acm.0000000000001263. 27332867 PMC5177544

[52] Whitworth A , Haining S , Stringer H. Enhancing research capacity across healthcare and higher education sectors: development and evaluation of an integrated model. BMC Health Serv Res. 2012;12:287. doi: 10.1186/1472-6963-12-287. 22929175 PMC3471044

[53] Kalet A , Lusk P , Rockfeld J , et al. The challenges, joys, and career satisfaction of women graduates of the robert wood johnson clinical scholars program 1973–2011. J Gen Intern Med. 2020;35(8):2258–2265. doi: 10.1007/s11606-020-05715-3. 32096079 PMC7403242

[54] Walters SJ , Stern C , Robertson-Malt S. The measurement of collaboration within healthcare settings: a systematic review of measurement properties of instruments. JBI Database System Rev Implement Rep. 2015;14(4):138–197. doi: 10.11124/jbisrir-2016-2159. 27532315

[55] Blanchard M , Burton MC , Geraci MW , et al. Best practices for physician-scientist training programs: recommendations from the alliance for academic internal medicine. Am J Med. 2018;131(5):578–584. doi: 10.1016/j.amjmed.2018.01.015. 29410155

[56] Hall KL , Feng AX , Moser RP , Stokols D , Taylor BK. Moving the science of team science forward: collaboration and creativity. Am J Prev Med. 2008;35(2 Suppl):S243–S249. doi: 10.1016/j.amepre.2008.05.007. 18619406 PMC3321548

[57] Mâsse LC , Moser RP , Stokols D , et al. Measuring collaboration and transdisciplinary integration in team science. Am J Prev Med. 2008;35(2):S151–S160. doi: 10.1016/j.amepre.2008.05.020. 18619395

[58] Hawk LW Jr. , Murphy TF , Hartmann KE , Burnett A , Maguin E. A randomized controlled trial of a team science intervention to enhance collaboration readiness and behavior among early career scholars in the clinical and translational science award network. J Clin Transl Sci. 2024;8(1):e6–33. doi: 10.1017/cts.2023.692. 38384923 PMC10877513

[59] Meagher E , Taylor L , Probsfield J , Fleming M. Evaluating research mentors working in the area of clinical translational science: a review of the literature. Clin Transl Sci. 2011;4(5):353–358. doi: 10.1111/j.1752-8062.2011.00317.x. 22029808 PMC3727275

[60] McRae M , Zimmerman KM. Identifying components of success within health sciences-focused mentoring programs through a review of the literature. Am J Pharm Educ. 2019;83(1):6976. doi: 10.5688/ajpe6976. 30894774 PMC6418850

[61] Yin HL , Gabrilove J , Jackson R , Sweeney C , Fair AM , Toto R. Sustaining the clinical and translational research workforce: training and empowering the next generation of investigators. Acad Med. 2015;90(7):861–865. doi: 10.1097/acm.0000000000000758. 26414054 PMC4587496

[62] Tsai AC , Ordóñez AE , Reus VI , Mathews CA. Eleven-year outcomes from an integrated residency program to train research psychiatrists. Acad Med. 2013;88(7):983–988. doi: 10.1097/ACM.0b013e318294f95d. 23702520 PMC3713635

[63] Feldon DF , Litson K , Jeong S , et al. Postdocs’ lab engagement predicts trajectories of phD students’ skill development. Proc Natl Acad Sci U S A. 2019;116(42):20910–20916. doi: 10.1073/pnas.1912488116. 31570599 PMC6800364

[64] Smyth SS , Coller BS , Jackson RD , Kern PA , McIntosh S , Meagher EA , et al. KL2 scholars’ perceptions of factors contributing to sustained translational science career success. J Clin Transl Sci. 2022;6(1):1–24. doi: 10.1017/cts.2021.886. PMC9003634 35433037

[65] Eder MM , Carter-Edwards L , Hurd TC , Rumala BB , Wallerstein N. A logic model for community engagement within the clinical and translational science awards consortium: Can we measure what we model? Acad Med. 2013;88(10):1430–1436. doi: 10.1097/ACM.0b013e31829b54ae. 23752038 PMC3784628

[66] Eder MM , Evans E , Funes M , et al. Defining and measuring community engagement and community-engaged research: clinical and translational science institutional practices. Prog Community Health Partnersh. 2018;12(2):145–156. doi: 10.1353/cpr.2018.0034. 30270224 PMC6237095

[67] Patten CA , Albertie ML , Chamie CA , et al. Addressing community health needs through community engagement research advisory boards. J Clin Transl Sci. 2019;3(2-3):125–128. doi: 10.1017/cts.2019.366. 31660236 PMC6802408

[68] Holzer J , Kass N. Understanding the supports of and challenges to community engagement in the CTSAs. CTS: Clinical & Translational Science. 2015;8(2):116–122. doi: 10.1111/cts.12205. 25196710 PMC4362794

[69] Buxton M , Hanney S. How can payback from health services research be assessed? J Health Serv Res Policy. 1996;1(1):35–43. 10180843

[70] Ziegahn L , Joosten Y , Nevarez L , et al. Collaboration and context in the design of community-engaged research training. Health Promot Pract. 2021;22(3):358–366. doi: 10.1177/1524839919894948. 31948272 PMC8612231

[71] Donovan C. State of the art in assessing research impact: introduction to a special issue. Res Evaluat. 2011;20(3):175–179. doi: 10.3152/095820211x13118583635918.

[72] Donovan C , Butler L , Butt AJ , Jones TH , Hanney SR. Evaluation of the impact of national breast cancer foundation-funded research. Med J Aust. 2014;200(4):214–218. doi: 10.5694/mja13.10798. 24580524

[73] Banzi R , Moja L , Pistotti V , Facchini A , Liberati A. Conceptual frameworks and empirical approaches used to assess the impact of health research: an overview of reviews. Health Res Policy Syst. 2011;9(1):26. doi: 10.1186/1478-4505-9-26. 21702930 PMC3141787

[74] Ari MD , Iskander J , Araujo J , et al. A science impact framework to measure impact beyond journal metrics. PLoS One. 2020;15(12):e0244407. doi: 10.1371/journal.pone.0244407. 33351845 PMC7755179

[75] Van Schyndel JL , Koontz S , McPherson S , et al. Faculty support for a culture of scholarship of discovery: a literature review. J Prof Nurs. 2019;35(6):480–490. doi: 10.1016/j.profnurs.2019.05.001. 31857059

[76] Monsura MP , Dizon RL , Tan CG Jr , Gapasin ARP. Why research matter?: an evaluative study of research productivity performance of the faculty members of the polytechnic university of the philippines. J Pharm Negat Results. 2022;13:680–694. doi: 10.47750/pnr.2022.13.S06.097.

[77] Ommering BWC , Dekker FW. Medical students’ intrinsic versus extrinsic motivation to engage in research as preparation for residency. Perspect Med Educ. 2017;6(6):366–368. doi: 10.1007/s40037-017-0388-3. 29170986 PMC5732111

[78] Pager S , Holden L , Golenko X. Motivators, enablers, and barriers to building allied health research capacity. J Multidiscip Healthc. 2012;5:53–59. doi: 10.2147/jmdh.S27638. 22396626 PMC3292402

[79] Emmons KM , Viswanath K , Colditz GA , Emmons KM , Viswanath K , Colditz GA. The role of transdisciplinary collaboration in translating and disseminating health research: lessons learned and exemplars of success. Am J Prev Med. 2008;35(2):S204–S210. doi: 10.1016/j.amepre.2008.05.009. 18619401

[80] Hays TC. The science of team science: commentary on measurements of scientific readiness. Am J Prev Med. 2008;35(2 Suppl):S193–S195. doi: 10.1016/j.amepre.2008.05.016. 18619399

[81] Heslin PA. Conceptualizing and evaluating career success. J Organ Behav. 2005;26(2):113–136. doi: 10.1002/job.270.

[82] Grant JS , Davis LL. Selection and use of content experts for instrument development. Res Nurs Health. 1997;20(3):269–274. doi: 10.1002/(sici)1098-240x(199706)20:. 9179180

[83] Almanasreh E , Moles R , Chen TF. Evaluation of methods used for estimating content validity. Res Social Adm Pharm. 2019;15(2):214–221. doi: 10.1016/j.sapharm.2018.03.066. 29606610

[84] Aromataris E , Munn Z. JBI manual for evidence synthesis. JBI;2021. (https://synthesismanual.jbi.global/) April. Accessed March 6, 2024.

[85] Polit DF , Yang F. Measurement and the measurement of change: A primer for health professionals. Lippincott Williams and Wilkins; 2016.

[86] Rosenkoetter U , Tate RL. Assessing features of psychometric assessment instruments: a comparison of the COSMIN checklist with other critical appraisal tools. Brain Impair. 2018;19(1):103–118. doi: 10.1017/BrImp.2017.29.

[87] Flake JK , Davidson IJ , Wong O , Pek J. Construct validity and the validity of replication studies: a systematic review. Am Psychol. 2022;77(4):576–588. doi: 10.1037/amp0001006. 35482669

[88] Hayes AF , Coutts JJ. Use omega rather than cronbach’s alpha for estimating reliability. But Commun Methods Meas. 2020;14(1):1–24. doi: 10.1080/19312458.2020.1718629.

[89] Malkewitz CP , Schwall P , Meesters C , Hardt J. Estimating reliability: a comparison of cronbach’s α, mcDonald’s ωt and the greatest lower bound. Soc Sci Humanit Open. 2023;7(1):100368. doi: 10.1016/j.ssaho.2022.100368.

[90] Orçan F. Comparison of cronbach’s alpha and mcdonald’s omega for ordinal data: are they different? Int J Assmt Tool Educ. 2023;10(4):709–722. doi: 10.21449/ijate.1271693.

[91] Taber KS. The use of cronbach’s alpha when developing and reporting research instruments in science education. Res Sci Educ. 2018;48(6):1273–1296. doi: 10.1007/s11165-016-9602-2.

[92] Polit DF. Getting serious about test-retest reliability: a critique of retest research and some recommendations. Qual Life Res. 2014;23(6):1713–1720. doi: 10.1007/s11136-014-0632-9. 24504622

