# Supplementary material for: The development, validity, and reliability of the Researcher Investment Tool
Source: J Clin Transl Sci. 2025 Aug 11;9(1):e160. doi: 10.1017/cts.2024.673 (PMC12392352; doi:10.1017/cts.2024.673)
Supplement: Joly et al. supplementary material [file S2059866124006733sup001.pdf]

NORTHERN NEW ENGLAND
CLINICAL & TRANSLATIONAL
RESEARCH NETWORK

Researcher Investment Tool

Section I: Researcher Experiences

- This section asks you to *share your level of experience* with a series of statements designed to assess your research skills, activities, productivity, collaboration, mentorship, community engagement, and research impact.
- Some items may be more relevant, given your research focus. If you have no experience with an item (regardless of reason), please check no experience.

Research Skills: The experiences investigators (at all levels) have in conducting activities related to research.
Please assess your *level of experience* with the following research skills and respond to each item in order for us to capture your full experience.

	No Experience	A Little Experience	Some Experience	A Lot of Experience	Extensive Experience
1.1. Designing a research project	☐	☐	☐	☐	☐
1.2. Writing a research proposal	☐	☐	☐	☐	☐
1.3. Applying for research funding	☐	☐	☐	☐	☐
1.4. Collaborating with other researchers, as a team member	☐	☐	☐	☐	☐
1.5. Leading a research team	☐	☐	☐	☐	☐
1.6. Writing a research application for IRB or IACUC	☐	☐	☐	☐	☐
1.7. Tracking the progress of a research study	☐	☐	☐	☐	☐
1.8. Managing research data	☐	☐	☐	☐	☐
1.9. Analyzing research data	☐	☐	☐	☐	☐
1.10. Delivering a research presentation	☐	☐	☐	☐	☐
1.11. Writing a research manuscript	☐	☐	☐	☐	☐
1.12. Closing a research study	☐	☐	☐	☐	☐

Service to Profession: The experience investigators (at all levels) have in participating in research-related service activities.
Please assess your *level of experience* with the following research-related service activities.

	No Experience	A Little Experience	Some Experience	A Lot of Experience	Extensive Experience
2.1. Leading a research facility or unit	☐	☐	☐	☐	☐
2.2. Teaching research skills	☐	☐	☐	☐	☐
2.3. Mentoring researchers	☐	☐	☐	☐	☐
2.4. Serving as a reviewer for a research manuscript	☐	☐	☐	☐	☐
2.5. Serving on an IRB/IACUC team	☐	☐	☐	☐	☐
2.6. Participating on a grant review team for any funder	☐	☐	☐	☐	☐
2.7. Participating in a NIH study section	☐	☐	☐	☐	☐
2.8. Serving on an editorial board for a peer-reviewed scientific journal	☐	☐	☐	☐	☐
2.9. Serving as a guest editor for a peer-reviewed scientific journal	☐	☐	☐	☐	☐

Service to the Profession Continued.

	No Experience	A Little Experience	Some Experience	A Lot of Experience	Extensive Experience
2.10. Presenting your research locally, by invitation (e.g. invited to be a panelist/keynote)	☐	☐	☐	☐	☐
2.11. Presenting your research nationally, by invitation (e.g. invited to be a panelist/keynote)	☐	☐	☐	☐	☐
2.12. Presenting your research internationally, by invitation (e.g. invited to be a panelist/keynote)	☐	☐	☐	☐	☐
2.13. Contributing to the development of new scientific guidelines	☐	☐	☐	☐	☐

Research Productivity: The experience investigators (at all levels) have in securing funding, contributing to scientific knowledge, and influencing future research, policies and practices.
Please assess your *level of experience* with the following research products and outcomes, regardless of your role.

	No Experience	A Little Experience	Some Experience	A Lot of Experience	Extensive Experience
3.1. Receiving research funding from internal/institutional sources	☐	☐	☐	☐	☐
3.2. Receiving research funding from external non-Federal sources	☐	☐	☐	☐	☐
3.3. Receiving research funding from industry sources	☐	☐	☐	☐	☐
3.4. Receiving research funding from external Federal sources (excluding NIH funding) (e.g., USDA, HRSA, AHRQ)	☐	☐	☐	☐	☐
3.5. Receiving research funding from NIH F grant (e.g., F31-F32)	☐	☐	☐	☐	☐
3.6. Receiving research funding from NIH early-career K grant (e.g., K01, K08, K23, K24)	☐	☐	☐	☐	☐
3.7. Receiving research funding from NIH R grant (e.g., RO3, R21, R01)	☐	☐	☐	☐	☐
3.8. Publishing a peer-reviewed manuscript	☐	☐	☐	☐	☐
3.9. Publishing research in a high impact journal	☐	☐	☐	☐	☐
3.10. Having research cited by other authors	☐	☐	☐	☐	☐
3.11. Receiving inquiries from other researchers about your work	☐	☐	☐	☐	☐
3.12. Translating research to the public using media (e.g., news articles, editorial, video interview)	☐	☐	☐	☐	☐
3.13. Having research findings lead to changes in policies/procedures in health care (e.g., coding)	☐	☐	☐	☐	☐
3.14. Having research findings influence best practices in care	☐	☐	☐	☐	☐
3.15. Having research findings impact basic clinical and translational processes	☐	☐	☐	☐	☐
3.16. Having research findings impact biomedical research progress	☐	☐	☐	☐	☐

Research Collaboration: The experience and activities investigators (at all levels) have working with different types of research teams.

Team Composition

Please assess your *level of experience* with the following research teams.

	No Experience	A Little Experience	Some Experience	A Lot of Experience	Extensive Experience
4.1. Participating in one or more multi-disciplinary research teams	☐	☐	☐	☐	☐
4.2. Participating in one or more multi-institutional research teams	☐	☐	☐	☐	☐
4.3. Leading multi-disciplinary research teams	☐	☐	☐	☐	☐
4.4. Leading multi-institutional research teams	☐	☐	☐	☐	☐
4.5. Participating in a research team that includes a patient voice	☐	☐	☐	☐	☐

Team Activities

Please assess your *level of experience* with the following team accomplishments.

	No Experience	A Little Experience	Some Experience	A Lot of Experience	Extensive Experience
5.1. Co-authoring research with a multi-disciplinary team	☐	☐	☐	☐	☐
5.2. Co-authoring research with a multi-institutional team	☐	☐	☐	☐	☐
5.3. Seeking out new partners (internal) to engage in research	☐	☐	☐	☐	☐
5.4. Seeking out new partners (multi-institutional) to engage in research	☐	☐	☐	☐	☐
5.5. Expanding research efforts to include cross-disciplinary approaches	☐	☐	☐	☐	☐
5.6. Collaborating with multiple researchers on grant proposals (e.g., multi-PI, co-investigator)	☐	☐	☐	☐	☐
5.7. Receiving multi-investigator grant funding	☐	☐	☐	☐	☐

Research Mentorship: The experience investigators (at all levels) have in receiving or providing individualized research mentoring.

Mentee Experience

Please assess your *level of experience* with the following mentee-related activities.

	No Experience	A Little Experience	Some Experience	A Lot of Experience	Extensive Experience
6.1. Receiving research mentorship (any type)	☐	☐	☐	☐	☐
6.2. Meeting with mentor(s) on a routine basis	☐	☐	☐	☐	☐
6.3. Creating a mentee/mentor plan	☐	☐	☐	☐	☐
6.4. Being introduced to colleagues in the field by mentor(s)	☐	☐	☐	☐	☐
6.5. Participating in research projects with mentors	☐	☐	☐	☐	☐
6.6. Gaining new skills from mentor(s)	☐	☐	☐	☐	☐
6.7. Developing a research career plan with mentor(s).	☐	☐	☐	☐	☐

Mentor Experience

Please assess your *level of experience* with the following mentor-related activities.

	No Experience	A Little Experience	Some Experience	A Lot of Experience	Extensive Experience
7.1. Mentoring students	☐	☐	☐	☐	☐
7.2. Mentoring junior researchers	☐	☐	☐	☐	☐
7.3. Assisting mentee(s) with their own grant proposals	☐	☐	☐	☐	☐
7.4. Assisting mentee(s) with their own funded projects	☐	☐	☐	☐	☐
7.5. Assisting mentee(s) with writing	☐	☐	☐	☐	☐
7.6. Advocating for compensated research time for mentee(s)	☐	☐	☐	☐	☐
7.7. Introducing mentee(s) to new research opportunities	☐	☐	☐	☐	☐
7.8. Introducing mentee(s) to colleagues in the field	☐	☐	☐	☐	☐

Community Engagement: The experience investigators (at all levels) have with community-engaged research efforts.

Please assess your *level of experience* with the following community engagement-related questions.

	No Experience	A Little Experience	Some Experience	A Lot of Experience	Extensive Experience
8.1. Reaching out to engage community partners in my research activities	☐	☐	☐	☐	☐
8.2. Identifying common research interests with the community	☐	☐	☐	☐	☐
8.3. Aligning my research interests with community priorities	☐	☐	☐	☐	☐
8.4. Participating in a research team that included a community voice	☐	☐	☐	☐	☐
8.5. Communicating research findings back to community partners	☐	☐	☐	☐	☐

Research Impact: The experience investigators (at all levels) have about the influence of their research efforts.

Please assess your *level of experience* related to the impact of your research.

	No Experience	A Little Experience	Some Experience	A Lot of Experience	Extensive Experience
9.1. Participating in research that has positively influenced health outcomes	☐	☐	☐	☐	☐
9.2. Participating in research that has positively influenced health policy	☐	☐	☐	☐	☐
9.3. Participating in research that has positively influenced the biomedical field	☐	☐	☐	☐	☐
9.4. Participating in research that has generated new theory or concepts	☐	☐	☐	☐	☐
9.5. Participating in research that has been translated to clinical practice	☐	☐	☐	☐	☐
9.6. Participating in research that has influenced the work of other researchers	☐	☐	☐	☐	☐

Section II: Researcher Perceptions

- This section asks you to *share your perceptions* based on a series of statements designed to assess institutional support.
- Please share your perceptions for all items.

Institutional Support: The perceptions investigators (at all levels) have regarding the culture, practices, and resources used by an organization to foster research.

Please rate your *perceptions* related to level of institutional support for research at your organization.

	Not At All	A Little	Some	A Lot	To a Great Extent
10.1. Leadership in my organization values my research	☐	☐	☐	☐	☐
10.2. My organization offers funding opportunities to support my research	☐	☐	☐	☐	☐
10.3. My organization promotes multi-disciplinary research (mine or others)	☐	☐	☐	☐	☐
10.4. My organization is committed to helping me build research skills	☐	☐	☐	☐	☐
10.5. My organization recognizes my research accomplishments	☐	☐	☐	☐	☐
10.6. My organization provides mentorship to support my career advancement	☐	☐	☐	☐	☐
10.7. My organization supports community-engaged research efforts	☐	☐	☐	☐	☐
10.8. I have been given designated time set aside for research	☐	☐	☐	☐	☐
10.9. I have the support I need from my organization to do my research	☐	☐	☐	☐	☐
10.10. I have the tools I need to do my research	☐	☐	☐	☐	☐
10.11. I am clear on the research expectations, given my current role	☐	☐	☐	☐	☐

Section III: Background

- This section asks you to *share your background and experience with research.* The items were designed to capture the characteristics of respondents who were engaged in a clinical and translational research initiative.
- This section is available upon request. Items in this section were not included in the psychometric testing.

Thank you for participating.
